# Intraoperative Neurophysiological Monitoring in Neurosurgery

**DOI:** 10.3390/jcm13102966

**Published:** 2024-05-17

**Authors:** Giusy Guzzi, Riccardo Antonio Ricciuti, Attilio Della Torre, Erica Lo Turco, Angelo Lavano, Federico Longhini, Domenico La Torre

**Affiliations:** 1 Neurosurgery Department, “R. Dulbecco” Hospital, 88100 Catanzaro, Italy; 2 Department of Medical and Surgical Sciences, “Magna Graecia” University of Catanzaro, 88100 Catanzaro, Italy; 3 Department of Neurosurgery, Belcolle Hospital, 01100 Viterbo, Italy; 4 Anesthesia and Intensive Care Unit, “R. Dulbecco” Hospital, 88100 Catanzaro, Italy

**Keywords:** intraoperative neurophysiological monitoring, neurosurgery, electrocorticography, electromyography, evoked potentials, direct cortical stimulation

## Abstract

Intraoperative neurophysiological monitoring (IONM) is a crucial advancement in neurosurgery, enhancing procedural safety and precision. This technique involves continuous real-time assessment of neurophysiological signals, aiding surgeons in timely interventions to protect neural structures. In addition to inherent limitations, IONM necessitates a detailed anesthetic plan for accurate signal recording. Given the growing importance of IONM in neurosurgery, we conducted a narrative review including the most relevant studies about the modalities and their application in different fields of neurosurgery. In particular, this review provides insights for all physicians and healthcare professionals unfamiliar with IONM, elucidating commonly used techniques in neurosurgery. In particular, it discusses the roles of IONM in various neurosurgical settings such as tumoral brain resection, neurovascular surgery, epilepsy surgery, spinal surgery, and peripheral nerve surgery. Furthermore, it offers an overview of the anesthesiologic strategies and limitations of techniques essential for the effective implementation of IONM.

## 1. Introduction

Intraoperative neurophysiological monitoring (IONM) is a real-time monitoring and assessment of the nervous system’s function and integrity during surgeries [1], used in a wide range of surgical procedures such as brain surgery [2], spinal surgery [3], vascular surgery [4,5], peripheral nerve surgery [6], orthopedic surgery [7,8], and otolaryngology procedures involving the nerves [9,10].

IONM necessitates a collective approach with a team comprising neurophysiologists, neurosurgeons, anesthesiologists, neurologists, and technologists, to set up and interpret monitoring equipment, analyze real-time data, and convey critical information to the surgical team [11,12]. It provides anatomical and functional information about the integrity of neural structures to the neurosurgeons, such as motor pathways, sensory pathways, and neural networks, improving the patient’s safety and post-operative outcomes [13].

The aim of this comprehensive narrative review is to provide the existing background information to physicians, including anesthesiologists, on the IONM techniques most frequently used during neurosurgery.

## 2. Methods

We have performed an electronic search of Medline from inception until 1 November 2023, with the restriction to adult-only patients and the English language. We utilized a combination of controlled vocabulary terms, text words, and keywords related to IONM techniques. Blocks of terms per concept were created. Two authors (GG and RAA) independently executed the search using keywords and their related MeSH terms such as “intraoperative neuromonitoring”, “neuromonitoring”, “neurosurgery”, “electrocorticography”, “stereo-electroencephalography”, “electromyography”, “somatosensory evoked potentials”, “motor evoked potential”, “direct cortical stimulation”, “brainstem auditory evoked potentials”, and “visual evoked potentials”. The constructed search strategy is reported in the Supplementary Materials.

Our review encompassed all English-language articles involving adult patients (aged > 18 years) undergoing various types of neurosurgeries (brain, spinal, or peripheral nerve) with any form of IONM. Additionally, studies elucidating neuromonitoring principles and techniques were considered for inclusion in this narrative review. Excluded were case reports, review articles, editorials, and studies available solely in abstract form. To identify any overlooked studies of relevance, we examined the references of included papers. Titles and abstracts were independently screened by two authors (GG and RAA) according to the inclusion criteria, and the full texts of potentially relevant reports were retrieved and examined. Any discrepancies were resolved through consultation with a third examiner (FL).

Given that our objective was to furnish a comprehensive narrative review aimed at informing physicians, including anesthesiologists, about IONM techniques commonly utilized during neurosurgery, we abstained from conducting pooled data analysis and assessing the risk of bias. The flow diagram detailing the article selection process is provided as Supplementary Materials (Figure S1).

## 3. IONM Techniques in Neurosurgery

The IONM techniques most frequently used during neurosurgery include (1) electrocorticography (ECoG) and stereo-electroencephalography (SEEG); (2) electromyography (EMG); (3) somatosensory evoked potentials (SSEPs); (4) motor evoked potential (MEP) and direct cortical stimulation (DCS); (5) brainstem auditory evoked potentials (BAEPs); and (6) visual evoked potentials (VEPs). The utilization of specific techniques in IONM allows for the observation and evaluation of distinct neural pathways, consequently reducing the necessity for intraoperative wake-up testing in patients vulnerable to neurological injury [14].

### 3.1. Electrocorticography (ECoG) and Stereo-Electroencephalography (SEEG)

**Figure 1:**
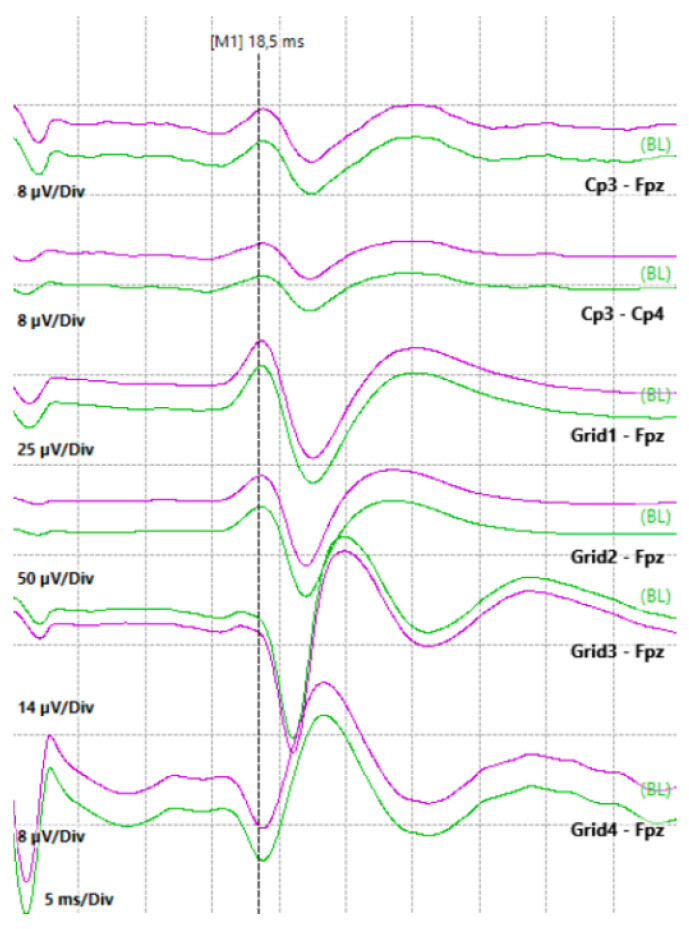
Phase reversal achieved by stimulating the right median nerve and recording at the cortical level with a subdural strip containing 4 contacts. The technique was used to identify the Rolandic sulcus, which in this case is identified by the phase reversal between contacts 3 and 4 of the strip.

Electrocorticography (ECoG) is a specific electroencephalographic technique which entails positioning electrode grids directly on the brain’s surface for neurophysiological analysis. These grids consist of multiple (4 to 32) electrodes strategically positioned to capture and analyze electrical activity from the cortical surface in real time with higher spatial and temporal resolution compared to non-invasive electroencephalography (EEG) [15]. ECoG allows the neurosurgery team to real-time identify and preserve essential areas responsible for various functions, including motor control, sensory perception, language, and epileptic activity [15,16,17]. For example, phase reversal (i.e., the point in time when the polarity of the recorded signal changes) represents the differentiation of the primary sensory areas by the motor areas of the brain (Figure 1). Furthermore, ECoG detects stimulation artifacts while stimulating the cortex, verifying that the stimulation is provided properly [18] and the occurrence of stimulation-induced afterdischarges and seizures [19]. Neurological effects seen with stimulation-associated afterdischarges or seizures may not be related to the cortical region stimulated; rather, they may be linked to the propagation of hypersynchronous neuronal activity to more distant sites [19]. Evidence of afterdischarges can alert the surgical team to the development of epileptic seizures, enabling them to employ preventive techniques such as irrigation of the cortex with cold water [20,21].

Stereo-electroencephalography (SEEG) is an invasive technique developed in the 1960s in France for the study of patients with drug-resistant epilepsy. The SEEG procedure involves multiple phases and is relatively complex, relying on the placement of intracranial electrodes via a stereotactic frame and a double grid system. The use of specialized implantation devices and the integration of multimodal neuroimaging techniques have further improved the methodology and clinical application of SEEG, reducing its complexity and enhancing safety [22,23]. The intracranial SEEG can precisely identify the epileptogenic zone and pinpoint the location of the “eloquent cortex” [22]. Hence, SEEG is an essential neuro-monitoring in cases when imaging is normal, noninvasive assessments show discrepancies, and a detailed mapping of cortical function is required due to the proximity of the presumed epileptogenic zone to the eloquent cortex, or in syndromes predisposed to multiple lesions [22].

### 3.2. Electromyography (EMG)

Electromyography (EMG) specifically evaluates the activity and functional integrity of somatic efferent nerves using subdermal or intramuscular electrodes. The procedure includes the depolarization of a motor nerve, triggering the generation of electrical potential within the innervated muscles [24].

One of the primary applications of EMG is the identification and preservation of peripheral nerves through its direct stimulation during peripheral nerve surgery [24,25,26]. Reduced muscle activity or abnormal EMG patterns serve as warning signs of potential nerve injury. EMG is also used in monitoring neuromuscular responses during neurosurgical procedures, such as resection of brain or spinal cord tumors [27]. Finally, EMG provides real-time feedback to the surgeon about the integrity of the nerve–muscle connection, specifically during nerve repair or grafting procedures [6].

### 3.3. Somatosensory Evoked Potentials (SSEPs)

**Figure 2:**
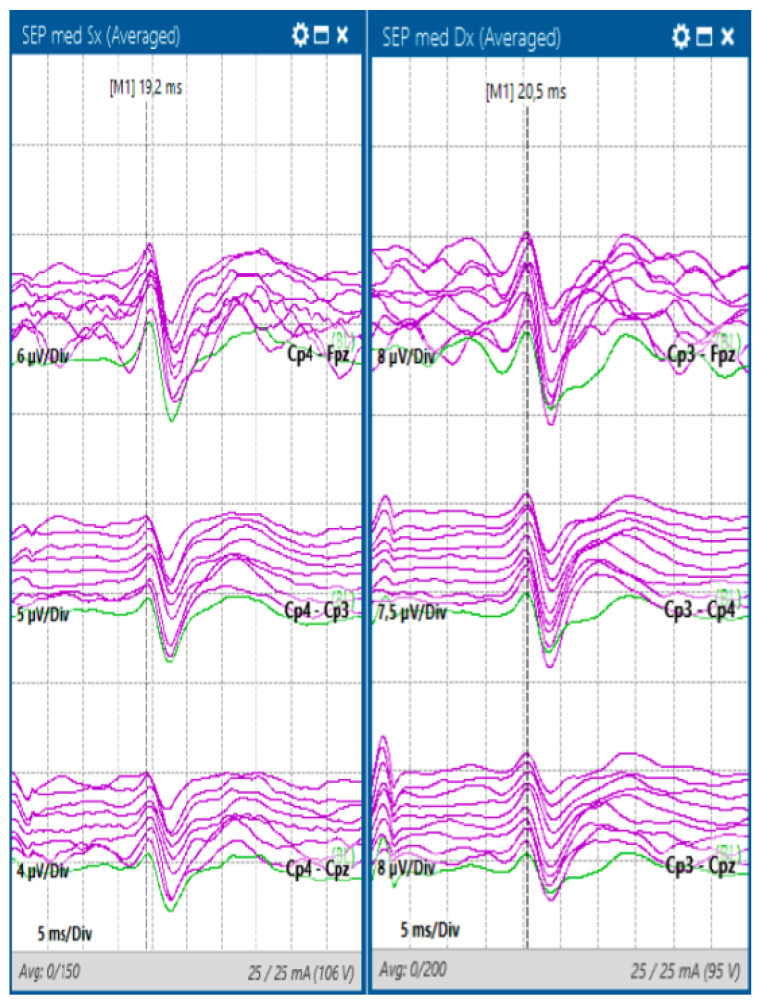
SSEPs of the upper limbs with stimulation from the median nerve using adhesive electrodes and recording from the scalp with corkscrew electrodes positioned according to the International 10–20 system.

Somatosensory evoked potentials (SSEPs) monitor the dorsal column–medial lemniscus pathway, which plays a role in tactile discrimination, vibration, and proprioception. The process involves stimulating sensory receptors in the skin, activating peripheral sensory nerves that extend through the nerve root to the ipsilateral dorsal root ganglia in the spinal levels [28]. These initial neurons project to form the fasciculi gracilis and cuneatus, transmitting impulses from the lower and upper extremities, respectively [28]. The first synapse occurs in the lower medulla, followed by the crossing over of the impulses at the brainstem level and the formation of the medial lemniscus. Subsequently, the impulse ascends to the contralateral thalamus, ultimately conveying information to the primary sensory cortex in the parietal lobe [28]. For the upper extremities, monitoring primarily focuses on the median and ulnar nerves (Figure 2), whereas for the lower extremities, it encompasses the posterior tibial and peroneal nerves.

The ability to detect any changes in SSEPs, such as alterations in amplitude, latency, or waveform, is crucial during surgery. These changes can indicate potential damage or compromise to the sensory pathways, signaling the need for immediate corrective measures. The traditional SSEP warning criteria, established in the 1970s, involved a >50% amplitude reduction or >10% latency prolongation from the baseline. However, these criteria have limitations as they overly focus on latency and do not account for baseline drift or reproducibility. To tackle this issue, an adaptive criterion has been suggested, taking into account visually noticeable amplitude reduction from recent pre-change values and exceeding variability, particularly in cases of sudden and localized changes [29].

### 3.4. Motor Evoked Potentials (MEPs) and Direct Cortical Stimulation (DCS)

**Figure 3:**
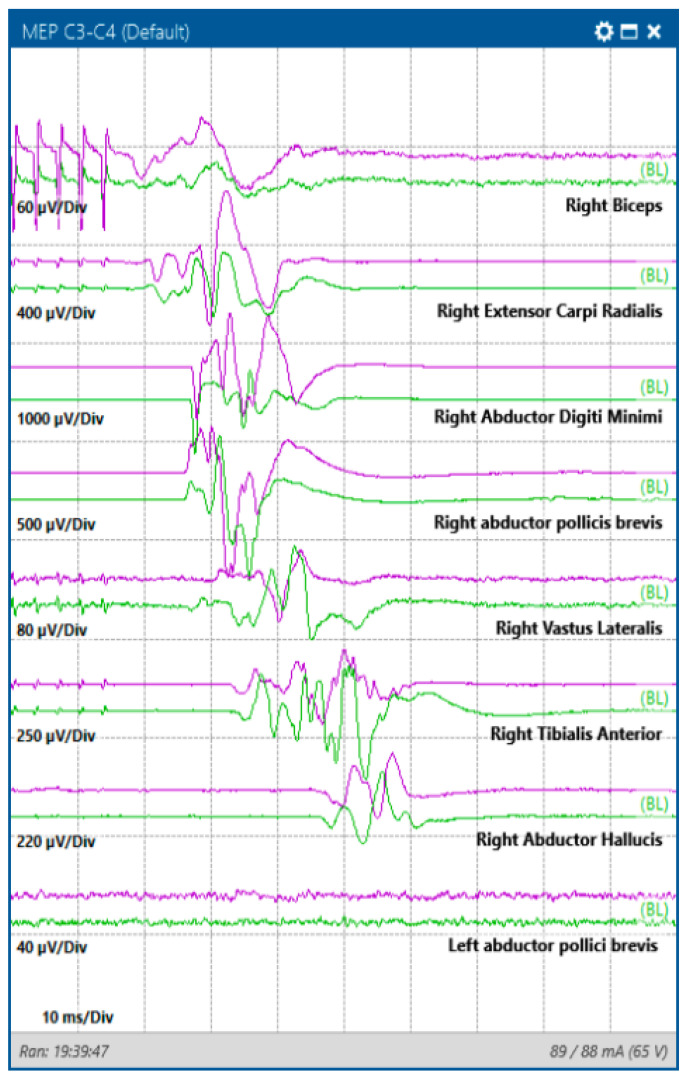
Transcranial motor evoked potentials (MEP) obtained by stimulating with corkscrew electrodes in the C3–FZ position at a threshold of 90 mA in a case of left parietal lesion.

Motor evoked potentials (MEPs) represent a specialized monitoring technique that concentrates on evaluating motor pathways within the nervous system. It is achieved through transcranial or direct electric stimulation of the motor cortex (DCS), inducing the excitation of corticospinal projections at different levels. The intensity of stimulation and the precise electrode placement are pivotal factors in pinpointing the specific brain areas where MEPs originate. Depending on the stimulation parameters, MEPs can be induced in various brain regions, such as the superficial white matter beneath the motor cortex, the deep white matter of the internal capsule, and the pyramidal decussation (Figure 3) [30].

Accurate investigation in supratentorial surgery with transcranial stimulation relies on carefully selecting the montage and stimulation intensity. The placement of scalp electrodes (C1, C2, Cz, C3, C4) follows the International 10–10 system coordinates. However, employing a lateral montage (C3/C4 or C4/C3 electrodes) with higher stimulation intensity may result in extensive stimulation across the entire motor pathway, from the motor cortex to the level of the foramen magnum. This has the potential to yield false negative results by stimulating the motor tract distal to a lesion [31,32]. Furthermore, such an approach may induce patient movement, which could impede the surgical procedure. Conversely, transcranial stimulation with inter-hemispheric (C1/C2 or C2/C1) or hemispheric (C3/Cz or C4/Cz) configurations, along with direct cortical stimulation (see below), allows for more targeted stimulation of the motor cortex [31,32].

The electrical potential generated during MEPs can be recorded at different locations, either directly at target muscles or from the epidural space at the spinal cord (d-waves). The latter is the best choice to monitor corticospinal tract integrity during spinal surgery [33,34]. The stability of the d-wave is a predictive indicator for favorable motor outcomes, even when the intraoperative transcranial MEPs are abolished or diminished. Noteworthy existing literature strongly advocates the simultaneous application of transcranial MEPs and d-waves in intramedullary spinal cord surgery. This approach aims to minimize false positives and provide enhanced guidance to the neurosurgeon during the surgical procedure [34,35,36,37].

**Figure 4:**
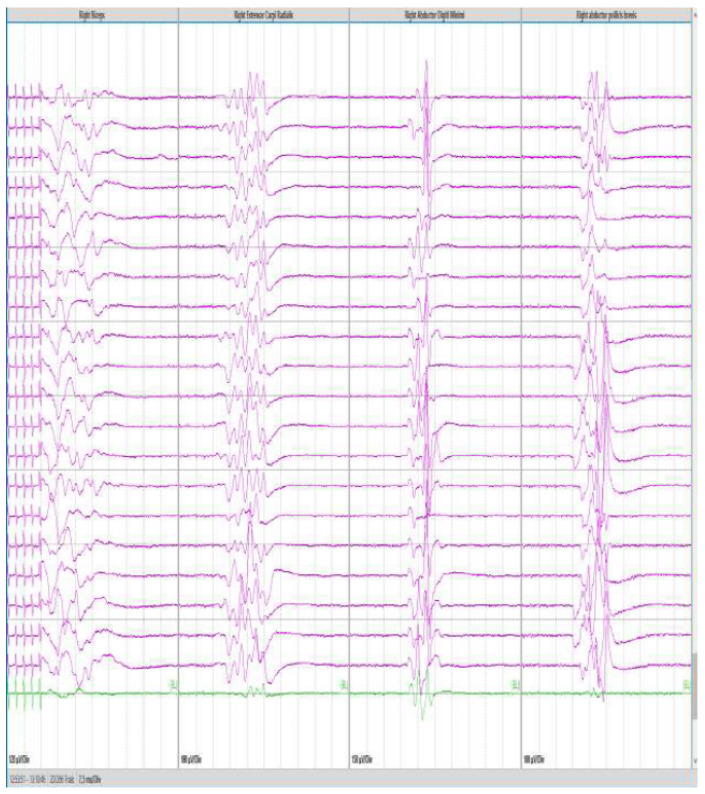
Direct cortical stimulation. Motor evoked potentials (MEP) obtained by stimulating from contacts on a cortical strip, positioned at the cortical level, with reference to a corkscrew electrode placed on the scalp. Panels from left to right show the responses obtained from the biceps muscle, the ulnar extensor of the wrist, the abductor of the fifth finger, and the short abductor of the thumb.

Direct cortical stimulation (DCS) is another technique that involves the application of electrical currents directly to the brain cortex. DCS involves placing some electrodes (generally a 6-contact strip) directly on the exposed cortical surface, in the subdural space, or inserted into the brain tissue. Electrical stimulation is delivered using a stimulator, and the responses are observed and recorded (Figure 4) [38]. Compared to MEPs, DCS necessitates less stimulation intensity and delivers highly localized and superficial motor cortex stimulation. DCS is often used to map the functional areas of the brain before surgical procedures. It can also be employed to identify the epileptic focus in patients with drug-resistant epilepsy or in some cases of awake brain surgery to allow the assessment of cognitive and language functions [38,39].

### 3.5. Brainstem Auditory Evoked Potentials (BAEPs)

Brainstem auditory evoked potentials (BAEPs)monitor the functionality of the auditory nerve and the auditory pathways within the brainstem. The auditory signal originates at the cochlear hair cell, where sound waves are converted into electrical signals. It then progresses through a series of anatomical structures, including the vestibulocochlear nerve, the superior olivary complex, the lateral lemniscus, the inferior colliculus, and the medial geniculate body. This sequential relay system guarantees the transmission of auditory information toward the primary auditory cortex, where it undergoes processing and interpretation.

BAEPs, consisting of seven distinct positive waves, are small auditory evoked potentials in response to an auditory stimulus, captured using A1 and A2 electrodes as the active points, while Cz or Fz is employed as the reference electrode positioned on the scalp [40]. It finds common usage in the surgical treatment of different pathologies involving the posterior cranial fossa and the cerebellopontine angle, such as acoustic neuroma, neurovascular compression syndrome, and brainstem tumor [41,42,43].

Within intraoperative BAEP alterations, the most frequently observed phenomenon is the latency change in wave V: a 50% reduction in the amplitude of wave V and a latency increase exceeding 0.5 ms serve as warning indicators. In addition, the absence of wave V indicates deafness [44,45]. In the case of cochlear ischemia secondary to compromise of the internal auditory artery, all components of BAEPs, including wave I, are affected. However, when there is a direct mechanical or thermal trauma to the eighth nerve, waves III and V may experience delays, attenuation, or even elimination, while wave I might remain unaffected [41,42,43,46].

Finally, a prolongation of the I-to-III interpeak interval, which is usually reversible, may occur when there is a stretching of the eighth nerve, in the process of scraping off tumors or during the retraction of the cerebellum or brainstem [42,46].

### 3.6. Visual Evoked Potentials (VEPs)

Visual evoked potentials (VEPs) assess the functional integrity of the optic pathways responsible for transmitting visual stimuli from the retina to the brain’s visual cortex in response to light. The process begins with the conversion of visual stimuli into nerve signals within the retina. Then, the nerve signals traverse through the optic nerve and reach the optic chiasma, where a partial crossing of fibers occurs. Subsequently, the signals continue their path through the optic tract, leading them to the lateral geniculate body, an important relay station. From there, the signals are further transmitted through the optic radiation, a bundle of nerve fibers, ultimately reaching their destination in the visual cortex located in the occipital lobe of the brain [47].

VEPs are captured through active electrodes (O1, O2, and Oz), referencing the vertex to the Cz reference electrode on the scalp over the occipital cortex in response to light stimuli [48]. When dealing with occipital brain lesions, subdural strip electrodes may be employed for recording [49,50,51]. Recent attention to VEP monitoring has been sparked by the growing utilization of simultaneous electroretinogram (ERG) recording, progress in light-emitting diodes (LEDs) manufacturing, and the adoption of white light stimulation [26,52].

To ensure adequate flash stimuli are delivered to the retina, ERG is recorded simultaneously with VEPs, as the dislocation of goggles could lead to inadequate stimulation of the retina. Nevertheless, VEPs may not be detectable in individuals with visual acuity below 0.1 and/or a visual field defect larger than hemianopsia. Initial findings showed that a correlation existed between postoperative declines in visual acuity and the absence of VEPs for a duration exceeding four minutes [53]. Nowadays, the most used warning criterion is a 50% decrement of the response in VEPs [26,54,55]. When white LEDs are used, an amplitude decrement > 20% could be considered an alarm criterion [52]. Interestingly, in the case of preoperative impaired visual function, VEPs may not be recorded in approximately 50% of cases, limiting their application in such cases [54]. In fact, in some studies, these patients have been excluded from the eligible population [56]. Although VEP has gained interest during neurosurgery to preserve the functional integrity of the optic system, further research is required to establish a more standardized warning criterion.

## 4. Clinical Applications of Neuromonitoring

IONM has revolutionized the field of neurosurgery by significantly enhancing the safety and precision of various neurosurgical procedures. Despite the enormous development of technologies applied to brain mapping and monitoring, IONM is still the gold standard. Whether it is intracranial tumor resection, neurovascular, epilepsy, or spinal surgery, IONM is used as a technique that allows surgeons to monitor and safeguard neural structures, thereby reducing the risk of complications and improving patient outcomes. Table 1 summarizes the IONM techniques with their aims and possible applications during neurosurgery.

### 4.1. IONM in Intracranial Tumor Resection

Intracranial tumor resection surgery aims to extract as much of the tumor mass as possible while minimizing damage to the surrounding healthy brain tissue. To achieve this purpose, the use of IONM techniques, including SEEG, SSEPs, MEPs, and cortical mapping, is essential to properly identify critical functional structures of the brain.

**Figure 5:**
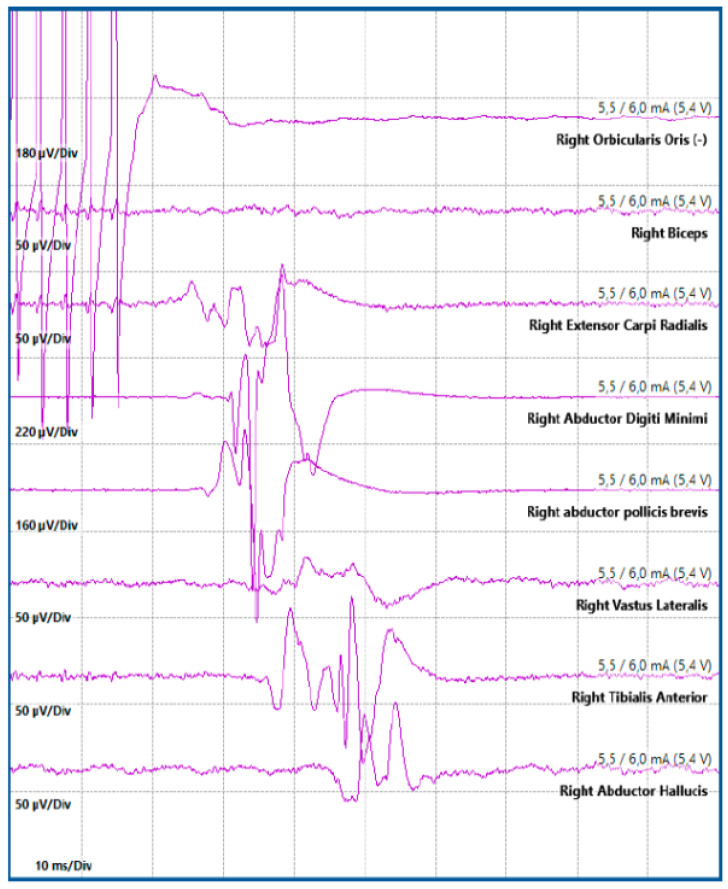
Subcortical stimulation. In a left parietal expansive lesion, during the removal phase, subcortical stimulation was performed with a suction probe, referenced to a corkscrew electrode placed on the scalp. Activation of the corticospinal tract was recorded at an intensity of 5.5 mA.

In the context of supratentorial tumors, IONM is a crucial component of modern neurosurgical procedures, particularly when removing tumors located near essential areas such as the Rolandic region and frontotemporal speech areas. To pinpoint the eloquent cortex and white matter tracts during various stages of tumor resection, the gold standard involves DCS and subcortical stimulation, facilitated by dedicated mono or bipolar forceps. These forceps generate a focused electric field, eliciting potentials on the cortex or subcortical bundles, which are distally recorded using various modalities, including EMG for motor function. This approach allows for the mapping of functional areas. A recent advancement involves the use of a new stainless steel suction for subcortical mapping (Figure 5). In a limited patient population undergoing brain surgery for tumor resection, this tool has demonstrated its ability to identify the corticospinal tract easily, ensuring the safety of surgical interventions in proximity to motor-eloquent structures [57,58].

To safeguard the corticospinal tract, an essential strategy includes ongoing MEP monitoring. This monitoring technique becomes especially crucial when dealing with the removal of tumors situated in or around the central region and insular tumors that extend deeply toward the internal capsule. The extent of tumor resection is determined by assessing the rate of MEP amplitude decrease, thereby ensuring the preservation of vital motor functions [26]. While MEPs predict deficits, their effectiveness as a warning sign is somewhat restricted, as signal alterations are only reversible in approximately 60% of cases [59]. Notably, irreversible motor deficits can manifest when direct cortical MEP undergoes changes during stimulation with a motor threshold of 1 mA. Conversely, it is advisable to halt tumor resection at the latest when reaching a motor threshold of 2 mA [59]. A recent study has reported that, in the immediate postoperative period, transcranial MEPs have sensitivity, specificity, positive predictive values, and negative predictive values equal to 17.5%, 100%, 100%, and 69.4%, respectively. In contrast, the direct cortical MEP monitoring showed the respective values of 25.0%, 100%, 100%, and 68.8%. It is worth mentioning that the sensitivity for predicting post-operative motor deficit at hospital discharge rose to 43.8% for transcranial MEPs and to 50.0% for direct cortical MEPs [60].

During an intracranial tumor resection of the motor area, some recently developed techniques, including the cortico-cortical evoked potentials (CCEPs) with subcortical evoked potentials (SCEPs), can be employed. This technique allows a continuous and dynamic mapping of white matter and subcortical pathways by a suction cannula coupled to a monopolar stimulator [61]. It is considered particularly precise and accurate, since the system stimulates the tissue at the removal site at low stimulation intensities (<5 mA). In addition, it has the advantage of continuously monitoring the neuronal pathways, whereas previous electrical stimulation techniques guaranteed only intermittent monitoring of the corticospinal tract [61].

Relying solely on sensory impairment is not considered a sufficient criterion to halt tumor resection [26]. In response to this concern, SSEPs are commonly employed as a supplementary method alongside MEPs monitoring during brain tumor surgeries [26]. The significance of SSEP monitoring becomes particularly pronounced in cases where the tumor involves vessels in the Sylvian fissure or during the trans-sylvian approach, as these scenarios may give rise to significant vasospasm [26]. Furthermore, VEP monitoring assumes a critical role in the resection of intrinsic brain lesions situated in close proximity to visual pathways and their associated regions, including temporal, temporo-insular, parietal, or parietooccipital lesions [26].

While IONM continues to play a crucial role in guiding brain surgeries for tumor resection, recent advancements in neuroimaging techniques (i.e., diffusion tensor imaging tractography and transcranial magnetic stimulation) have sparked a renewed focus on preoperative mapping. This approach enables neurosurgeons to identify and analyze the neural connectivity responsible for motor and language functions. Preoperative mapping has demonstrated a strong correlation with the findings obtained during surgery using direct cortical and subcortical mapping techniques [62,63,64,65].

### 4.2. IONM in Neurovascular Surgery

IONM is also employed in vascular surgeries involving aneurysms, arteriovenous malformations, and cavernous malformations. These procedures may be complicated by the occurrence of cerebral ischemia. In this field of neurosurgery, IONM can identify impending ischemia in the vascular territories of interest, enabling adjustments in intraoperative management to prevent ischemic stroke.

Among the available techniques that can be employed, cerebral blood flow monitoring, cerebral oxygenation, SEEG, and ECoG are of particular interest. Current intraoperative tools to monitor the cerebral blood flow include indocyanine green angiography [66,67,68], Doppler and transit-time ultrasound [69,70,71,72], and percutaneous transfemoral digital subtraction angiography [73,74,75]. Recently, a new tool called laser speckle contrast imaging (LSCI) has been developed for monitoring intraoperative cerebral blood flow [76]. This hardware is attached to the surgical microscope, allowing continuous real-time monitoring of cerebral blood flow [76]. Numerous studies have already showcased the potential of LSCI in human neurosurgical procedures, demonstrating its effectiveness as a cerebral blood flow monitoring tool, including its application during neurovascular surgery [76,77,78,79].

Cerebral oxygenation can also be monitored using the quantitative frequency-domain near-infrared spectroscopy (Q-NIRS). By measuring a reduction of tissue oxyhemoglobin and brain tissue oxygen saturation, along with an increase in deoxyhemoglobin, Q-NIRS can effectively detect an ischemic event [80].

When utilizing ECoG, the H-beta waveform proves highly specific and serves as an indicator of arterial occlusion. This waveform exhibits early, high-frequency waves (HF-Beta3, 23–37 Hz), reflecting the transition from aerobic to anaerobic metabolism due to reduced blood flow. These waves signify initial reversible neuronal damage. If ischemia persists, the appearance of delta-theta waves (2–6 Hz) follows, eventually leading to focal burst suppression patterns [81]. The shift to delta waves or focal burst suppression is linked with irreversible parenchymal damage [82]. Nonetheless, some authors have raised questions about the sensitivity of ECoG in detecting abnormalities in deeply seated lesions, such as those in the basal ganglia and internal capsule. In contrast, EEG may not be sufficiently sensitive to capture early HF-beta3 changes indicative of initial ischemic changes, which ECoG can discern [83,84].

Finally, SSEPs boast a well-established history in cerebral aneurysms, dating back to the 1980s [85]. Subsequently, other monitoring systems such as MEPs, BAEPs, and VEPs have been implemented based on the location of the vascular lesion and the potential surgical risks.

### 4.3. IONM in Epilepsy Surgery

Despite being the mainstay of epilepsy treatment, antiepileptic drug therapy is ineffective or not tolerated in 30% of patients. For these patients, epilepsy surgery could be a valid alternative for both lesional and nonlesional cases [86].

In addition to the existing neuroimaging techniques employed to guide the neurosurgeon during the procedure [87], IONM serves as an additional tool, particularly in cases of extratemporal epilepsy and nonlesional epilepsy closely associated with particular functional brain regions [11,87].

In epilepsy surgery, ECoG assesses interictal discharges, delineates functional areas, and facilitates the recording and localization of ictal activity. Depth electrodes, often combined with subdural cortical grids, are used to detect ictal or interictal events in subcortical regions during perioperative neuromonitoring [87]. ECoG can identify additional electrically active foci beyond the lesion, improving the 3-year seizure-free rate in patients undergoing both lesionectomy and spike-positive tissue resection compared to those undergoing only lesionectomy. Furthermore, ECoG likely reduces the reoperation rates and overall surgical morbidity in extralesional resections [88].

DCS is also applied in patients undergoing both lesional and nonlesional procedures for extratemporal epilepsy, particularly in areas close to motor, somatosensory, or language pathways. It is important to highlight that language functions, including repetition and comprehension, require testing while patients are awake [89]. Additionally, CCEP with SCEP can be utilized to identify seizure propagation pathways and monitor the cortical motor network and dorsal language pathway [90].

Awake surgery offers some advantages for epilepsy surgery. Firstly, it allows for intraoperative functional mapping under conscious conditions, enabling precise identification of crucial brain areas [89]. Secondly, intraoperative ECoG can be obtained without the influence of anesthesia, offering comparable information to chronic invasive ECoG recording in the interictal phase [91]. However, awake surgery presents certain drawbacks, including spatial constraints of craniotomy, limited intraoperative time, and the inability to capture ictal events. Moreover, the interpretation of ECoG findings remains contentious [17,92]. In addition, as recently reported by a systematic review, awake neurosurgery for epilepsy is complicated by intraoperative seizures in approximately 22% of cases [93]. Another challenge is patient cooperation, as some individuals, particularly children, or those with intellectual disabilities or psychiatric issues may struggle to participate fully in awake surgery [94]. Therefore, the choice of the anesthesiologic strategy should be dependent on patient-based criteria.

SEEG also records ictal electrical activity in predefined cortical targets for subsequent ablation of the presumed epileptogenic zone. SEEG covers extensive bilateral hemispheres with precise sampling from sulcal areas and deep brain structures. A hybrid technique combining SEEG and subdural strip electrode placement may address SEEG limitations. Improved electrode implantation accuracy and safety result from three-dimensional angiography, frameless MRI, advanced multimodal planning, and robot-assisted implantation [95].

Moreover, in current practice, SEEG is used not only for the identification of epileptogenic zones but also as a therapeutic tool, i.e., thermocoagulation [22,95].

### 4.4. IONM in Spinal Surgery

Spine surgery inherently carries a risk of causing harm to critical neural structures, with neurological complications ranging from 1.3% to 31% [96]. These complications may arise from direct mechanical forces on the spinal cord and indirect ischemic changes during corrective maneuvers [97,98]. To mitigate these risks, neurosurgeons employ monitoring techniques such as MEPs and SSEPs to safeguard spinal cord integrity [99,100].

**Figure 6:**
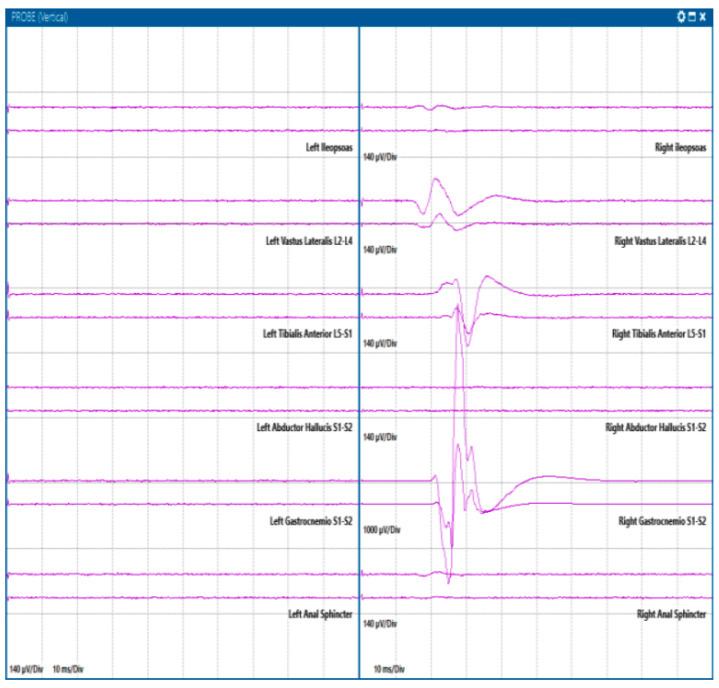
Motor evoked potentials in spinal surgery. The figure reports the MEP obtained, from top to bottom, in the left and right iliopsoas, vastus lateralis, tibialis anterior, abductor hallucis, gastrocnemius, and anal sphincter in a case of cauda surgery. The stimulation was obtained with a bipolar probe at 0.4 mA.

Figure 6 is an example of the application of MEPs during spinal surgery. The safety threshold for MEP reduction in spinal surgery varies. Langeloo et al. identified an amplitude reduction of 80% or more as a reliable criterion for potential impending neurological deficits [101]. Kobayashi et al. suggested a 70% reduction alert threshold, especially in procedures involving spinal deformities and tumors [36]. Pelosi et al. used a criterion of over 50% reduction in baseline amplitudes, combining MEPs with SSEPs for safer and more reliable monitoring in spinal deformity surgeries [102].

In anterior cervical spinal cord surgery, SSEPs are the most commonly used IONM technique (99.9%), followed by EMG (81.3%) and MEPs (64.8%) [103]. However, the value of monitoring during anterior cervical discectomy and fusion surgery is questionable due to the high incidence of false positives. Conversely, the simultaneous use of intraoperative MEPs and SSEPs is preferred during posterior approaches for cervical spondylotic myelopathy surgeries [104,105,106,107,108].

In a prospective multicenter study, 1156 cases of thoracic spine surgeries were monitored using transcranial MEPs, with threshold alerts set at an amplitude reduction exceeding 70% from the baseline. The study demonstrated a remarkable overall sensitivity of 91.9% and specificity of 88.4% in predicting positive post-operative outcomes. Consequently, the authors strongly recommended the adoption of transcranial MEPs, emphasizing their utility, especially for patients with preexisting motor deficits before surgery [109].

The role of IONM in intradural extramedullary tumors remains debated. While MEPs may not be deemed essential, recent findings suggest SSEPs significantly contribute to neurological preservation [110]. Further research is needed to assess the feasibility and significance of the D-wave technique in this surgery [110]. Conversely, for intramedullary spinal cord tumors, combining dorsal column mapping and spinal cord stimulation for SSEPs can be useful in identifying the anatomical midline, often distorted by the tumor’s anatomy [111]. A systematic review and meta-analysis proved that MEPs are characterized by the best specificity, whereas SSEPs show the greatest sensitivity in predicting postoperative neurological outcomes. However, a combination of different modalities of IONM resulted in the best diagnostic tool during surgery for the resection of intramedullary spinal cord tumors [112]. The same group of authors also reported that IONM is characterized by a high sensitivity and specificity in predicting the postoperative neurological outcomes at 6 weeks in patients undergoing resection of intradural extramedullary spinal cord tumor [113]; however, the incidence of false positives may influence the surgeon to continue or stop the surgical procedure. These cases are characterized by a high rate of tumor recurrence (around 90%) at the magnetic resonance imaging after one year [113]. Therefore, before making a decision, the surgical team, including the anesthesiologist, must check if there are any confounding factors, such as technical artifacts, an inappropriate anesthesiologic plan, hemodynamic instability, and muscular activity [113].

In patients with traumatic spinal cord injury, the use of transcranial MEPs has also been investigated [114]. Given the low prevalence of neurological complications (2.3%) and the low positive predictive value (18.4%), single usage of transcranial MEP monitoring during traumatic spinal injury surgery has not been recommended. It remains to be understood if the application of a multimodal IONM may improve the positive predictive value in this scenario [114].

Indeed, several recent studies have yielded inconclusive evidence regarding the improvement of neurological outcomes when utilizing IONM in various spinal cord surgical procedures, including anterior cervical discectomy and fusion surgery [115], thoracolumbar spine surgery [116], and surgeries addressing tethered cord syndrome or spinal intradural tumors [117]. A specific investigation involving 26 patients undergoing intramedullary spinal ependymoma surgery underscored a considerable rate of both false-positive and false-negative results associated with the use of IONM methods [118]. In light of these recent discordant findings, many experts are advocating for caution against the indiscriminate use of IONM [119], and the scarcity of robust evidence has resulted in a lack of definitive consensus or evidence-based guidelines for the secure and effective integration of IONM during these procedures [3,120]. While the routine use of EMG and SSEPs alone during spine surgery has lost support in the literature, the adoption of a multimodal IONM approach, including EMG, SSEPs, and MEPs, is frequently deemed necessary [121,122].

### 4.5. IONM in Peripheral Nerve Surgery

IONM plays a crucial role in enhancing assessments of the peripheral nervous system across various surgical procedures, improving decision-making, and leading to better surgical outcomes. When combined with neuroimaging, IONM assists in pinpointing different peripheral nerve pathologies, documenting structural nerve alterations such as swelling, compression, tumors, or injuries, and uncovering underlying issues [123,124].

During surgical interventions on peripheral nerves, IONM is frequently used to monitor all peripheral nerves, including the brachial plexus, lumbosacral plexus, and spinal roots, providing real-time, essential information to the surgical team and guiding surgeons in taking the most appropriate interventions [125,126,127,128]. It is now standard practice in surgeries for various conditions, such as entrapment neuropathies, traumatic nerve injuries and repairs, peripheral nerve tumors, and any surgical procedure posing a risk of peripheral nerve damage [129,130,131,132].

The two most utilized techniques are triggered EMG and nerve action potentials (NAPs). Triggered EMG involves electrically stimulating a nerve using a handheld bipolar stimulator, while simultaneously recording the EMG signal in the innervated muscular group using paired needle electrodes. This method enables the comprehensive monitoring of the entire motor pathway, from the stimulation point to the muscles, encompassing the nerve and neuromuscular junction. Despite its ease of use, triggered EMG is sensitive to anesthetic drugs such as muscle relaxants. Additionally, the signal amplitude varies significantly based on muscular trophism, potentially limiting its ability to accurately represent neural integrity and ultimate functional recovery [133].

NAPs, in contrast, are acquired using a pair of bipolar probes positioned directly on the nerve, with one probe stimulating and the other recording the resulting signal, maintaining a minimal distance of 5 cm to minimize artifacts [134]. In reconstructive surgery, NAPs serve to pinpoint the precise location of neural lesions, unaffected by anesthetic drugs, including neuromuscular blocking agents. Moreover, during surgeries for peripheral nerve tumors, the proximal margin of the lesion is readily discernible through a decrease in signal amplitude, while the distal point can only be identified clinically [133].

## 5. Anesthesia during IONM

During neurosurgery that involves the use of IONM, careful consideration must be given to selecting the optimal anesthetic plan [135].

The sensitivity and accuracy of collected neurophysiological data are significantly influenced by the chosen anesthetic technique. All anesthetic medications exert some level of interference with evoked potentials and thus need to be maintained at consistent levels throughout the surgery. The use of intravenous bolus infusions or abrupt alterations in the minimum alveolar concentrations of inhaled anesthetics could potentially compromise the precision of signal measurements [135]. For reliable evoked potential measurement, it is essential to maintain stable alveolar and serum concentrations of the anesthetic agents. Achieving this is most effectively done through continuous intravenous infusions of anesthetic agents. Currently, the preferred method in IONM is total intravenous anesthesia (TIVA) or target-controlled infusion (TCI) without the use of neuromuscular blocking agents. In particular, the preferred hypnotic agent is propofol, with remifentanil or sufentanil as analgesic drugs [135]. In recent times, the utilization of low doses of dexmedetomidine has emerged as a viable option as an adjuvant in neuroanesthesia. This is attributed to its dual benefits of providing analgesia and reducing the need for other anesthetic agents while aiding IONM [136].

Very recently, remimazolam has been introduced in clinical practice. Remimazolam is a new ultrashort-acting benzodiazepine with high water solubility and metabolism via tissue esterases [137]. Few reports exist on the use of remimazolam during IONM. In healthy volunteers, remimazolam induces at the EEG an initial increase in beta activity and a late increase in the delta frequency band [138]. The increase in beta activity may explain why some patients could not reach deep sedation with remimazolam, as monitored with EEG-derived indexes (see below) [139]. Remimazolam has also been shown to not interfere with the SSEPs, VEPs, and MEPs [140,141].

Neuromuscular blocking agents cannot be used since they interfere with EMG and MEPs if administered close to the IONM assessment [142,143]. However, rocuronium, a non-depolarizing neuromuscular blocking agent, could be used at low doses (0.3 mg·kg) at induction of anesthesia, to facilitate airways management, laryngoscopy, and tracheal intubation [9]. This dose is the effective dose (ED_95_), as it induces 95% depression of muscle contraction within 3 to 5 min and it secures 25% recovery of the motor response within 30 min [9,144,145]. In addition, rocuronium has the advantage of a specific antidote (i.e., sugammadex), which can be administered to immediately revert the neuromuscular block [146].

Monitoring the depth of anesthesia is also crucial during surgeries, particularly when IONM is employed. If sedation is too light, the patient might inadvertently move during the procedure, which could disrupt the surgical process and compromise patient safety. On the other hand, excessive sedation can potentially dampen the signals from IONM, affecting the quality of the collected data [147]. To attain the desired depth of anesthesia, brain function monitors are utilized. These monitors provide the depth of sedation by analyzing the EEG signal from a small number of frontal electrodes and generate a numerical scale ranging from 0 (indicating burst suppression) to 100 (indicating a fully awake patient). Typically, the optimal range of sedation falls between 40 and 60 on this scale [148,149].

Finally, other factors, such as hemodynamic stability, and other variables (changes in glycemia, electrolytes, gas exchange, hypothermia, reduced circulating blood volume and cerebral blood flow, and increased pressure in the superior vena cava) could potentially disrupt the accurate capture of signals during IONM procedures and may provide misleading information to the surgical team [135,150,151].

## 6. Limitations of IONM

When employing IONM, it is imperative to acknowledge potential limitations that may contribute to an elevated rate of false positives. These limitations may stem from a variety of factors, such as spontaneous muscular activity or twitches, electromagnetic interference, technical artifacts, the influence of anesthetic drugs, hemodynamic instability leading to hypoperfusion, hypothermia, patient movement, or positional changes during surgery. The influence of anesthetic drugs, hypoperfusion, and hypothermia have already been addressed above.

The misinterpretation of electrical activity originating from muscles as neural activity is a significant challenge during surgical procedures where precise SSEP monitoring of neural structures is needed. This artifact is exacerbated by the proximity of muscles to the nerves under surveillance, as the electrical signals generated by muscle contractions can inadvertently interfere with the monitoring process [152,153]. Although improving the quality of SSEP monitoring [154], the use of neuromuscular blocking agents is not possible if multimodal IONM, including MEPs, DCS, or EMG, is used [142,143].

Artifacts can be generated by electromagnetic interference or technical artifacts related to inadequate grounding, insulation of monitoring electrodes, or the presence of wireless medical equipment in the operating room [155].

Another technical artifact is the potential crossover that may occur during MEPs [156]. Crossover is a frequent phenomenon occurring when cortical stimulation induces activation of ipsilateral motor evoked responses [156]. In brain surgery, the presence of cross-activation presents a significant challenge as neural structures are activated distally from the area of interest. Addressing crossover issues may entail activating the motor pathway proximal to the surgical site, potentially mitigating the occurrence of false-negative responses [156].

Another issue that must be considered is the change in patient positioning or loss of signal. Patients undergoing spinal surgery are anesthetized in the supine position and afterward prone-positioned for the surgery. In this population, up to 43% of patients may face signal alterations, and in up to 10% of patients, IONM may severely attenuate or even lose the signals [157]. During cervical spine surgery, the rate of IONM loss of signal is lower (around 3%); in most cases, the sole patient repositioning caused a complete restoration of potentials [158].

These concerns could result in misunderstandings and unnecessary alerts, potentially steering the surgical team off course during the neurosurgical procedure. Hence, precise electrode positioning, effective insulation methods, a comprehensive understanding of techniques, a well-planned anesthesia strategy, and careful patient positioning are imperative to reduce the likelihood of false-positive outcomes.

## 7. Conclusions

The use of IONM is rapidly gaining importance during neurosurgical procedures. The selection of techniques to be used is closely connected to the type of surgery and should be personalized on a case-by-case basis. Due to its significant role in reducing post-operative deficits and improving patient outcomes, IONM should be integrated on an individual choice basis. This integration should be accompanied by specific training to ensure the optimal selection and application of techniques, and to reduce false-positive results.

## Figures and Tables

**Table 1:** Intraoperative neuromonitoring techniques and possible applications.

	Aim of the Technique	Evidence-Based Indications in Neurosurgery
*Electro-corticography (ECoG)*	Identification and preservation of cerebral cortical areas	Neurovascular surgery, epilepsy surgery
*Stereo-electroencephalography (SEEG)*	Identification of epileptogenic zones and the “eloquent cortex”	Intracranial tumor resection, neurovascular surgery, epilepsy surgery
*Electromyography (EMG)*	Identification and preservation of peripheral nerves	Spinal surgery, peripheral nerve surgery
*Somatosensory evoked potentials (SSEPs)*	Warning of potential damage to the sensory pathways	Intracranial tumor resection, spinal surgery
*Motor evoked potentials (MEPs)*	Evaluation of the motor pathways	Intracranial tumor resection, spinal surgery
*Direct cortical stimulation (DCS)*	Evaluation of the motor pathways through direct stimulation of the cortex	Intracranial tumor resection, epilepsy surgery
*Brainstem auditory evoked potentials (BAEPs)*	Monitoring the functionality of the auditory nerve and the auditory pathways within the brainstem	Intracranial tumor resection
*Visual evoked potentials (VEPs)*	Assessing the functional integrity of the optic pathways	Intracranial tumor resection

## Data Availability

Not applicable.

## Supplementary Materials

The following supporting information can be downloaded at: https://www.mdpi.com/article/10.3390/jcm13102966/s1, Search strategy; Figure S1: Flow diagram.

## References

[1] Grosland J.O., Todd M.M., Goldstein P.A. (2018). Neuromonitoring in the ambulatory anesthesia setting: A pro-con discussion. Curr. Opin. Anaesthesiol..

[2] Zebian B., Vergani F., Lavrador J.P., Mukherjee S., Kitchen W.J., Stagno V., Chamilos C., Pettorini B., Mallucci C. (2017). Recent technological advances in pediatric brain tumor surgery. CNS Oncol..

[3] Lall R.R., Hauptman J.S., Munoz C., Cybulski G.R., Koski T., Ganju A., Fessler R.G., Smith Z.A. (2012). Intraoperative neurophysiological monitoring in spine surgery: Indications, efficacy, and role of the preoperative checklist. Neurosurg. Focus.

[4] Moehl K., Shandal V., Anetakis K., Paras S., Mina A., Crammond D., Balzer J., Thirumala P.D. (2022). Predicting transient ischemic attack after carotid endarterectomy: The role of intraoperative neurophysiological monitoring. Clin. Neurophysiol..

[5] Szelenyi A., Fernandez-Conejero I., Kodama K. (2022). Surgery and intraoperative neurophysiologic monitoring for aneurysm clipping. Handb. Clin. Neurol..

[6] Strommen J.A., Skinner S., Crum B.A. (2022). Neurophysiology during peripheral nerve surgery. Handb. Clin. Neurol..

[7] Liu W., Li Y., Qiu J., Shi B., Liu Z., Sun X., Zhu Z., Qiu Y. (2023). Intra-operative neurophysiological monitoring in patients with thoracic spinal stenosis: Its feasibility and high-risk factors for new neurological deficit. Orthop. Surg..

[8] Jahangiri F.R., Blaylock J., Qadir N., Ramsey J.A. (2020). Multimodality intraoperative neurophysiological monitoring (ionm) during shoulder surgeries. Neur. J..

[9] Garofalo E., Bruni A., Scalzi G., Curto L.S., Rovida S., Brescia V., Gervasi R., Navalesi P., Innaro N., Longhini F. (2021). Low-dose of rocuronium during thyroid surgery: Effects on intraoperative nerve-monitoring and intubation. J. Surg. Res..

[10] Edwards B.M., Kileny P.R. (2005). Intraoperative neurophysiologic monitoring: Indications and techniques for common procedures in otolaryngology-head and neck surgery. Otolaryngol. Clin. N. Am..

[11] Timoney N., Rutka J.T. (2017). Recent advances in epilepsy surgery and achieving best outcomes using high-frequency oscillations, diffusion tensor imaging, magnetoencephalography, intraoperative neuromonitoring, focal cortical dysplasia, and bottom of sulcus dysplasia. Neurosurgery.

[12] Epstein N.E. (2014). Multidisciplinary in-hospital teams improve patient outcomes: A review. Surg. Neurol. Int..

[13] Tomasello F., Angileri F.F., Conti A., Scibilia A., Cardali S., La Torre D., Germano A. (2019). Petrosal meningiomas: Factors affecting outcome and the role of intraoperative multimodal assistance to microsurgery. Neurosurgery.

[14] Gunter A., Ruskin K.J. (2016). Intraoperative neurophysiologic monitoring: Utility and anesthetic implications. Curr. Opin. Anaesthesiol..

[15] Simon M.V., Nuwer M.R., Szelenyi A. (2022). Electroencephalography, electrocorticography, and cortical stimulation techniques. Handb. Clin. Neurol..

[16] Cuisenier P., Testud B., Minotti L., El Bouzaidi Tiali S., Martineau L., Job A.S., Trebuchon A., Deman P., Bhattacharjee M., Hoffmann D. (2020). Relationship between direct cortical stimulation and induced high-frequency activity for language mapping during seeg recording. J. Neurosurg..

[17] Fernandez I.S., Loddenkemper T. (2013). Electrocorticography for seizure foci mapping in epilepsy surgery. J. Clin. Neurophysiol..

[18] Crowther L.J., Brunner P., Kapeller C., Guger C., Kamada K., Bunch M.E., Frawley B.K., Lynch T.M., Ritaccio A.L., Schalk G. (2019). A quantitative method for evaluating cortical responses to electrical stimulation. J. Neurosci. Methods.

[19] Kalamangalam G.P., Tandon N., Slater J.D. (2014). Dynamic mechanisms underlying afterdischarge: A human subdural recording study. Clin. Neurophysiol..

[20] Nossek E., Matot I., Shahar T., Barzilai O., Rapoport Y., Gonen T., Sela G., Grossman R., Korn A., Hayat D. (2013). Intraoperative seizures during awake craniotomy: Incidence and consequences: Analysis of 477 patients. Neurosurgery.

[21] Sartorius C.J., Berger M.S. (1998). Rapid termination of intraoperative stimulation-evoked seizures with application of cold ringer’s lactate to the cortex. Technical note. J. Neurosurg..

[22] Iida K., Otsubo H. (2017). Stereoelectroencephalography: Indication and efficacy. Neurol. Med. Chir..

[23] Tomasello F., Conti A., La Torre D. (2016). 3d printing in neurosurgery. World Neurosurg..

[24] Ghatol D., Widrich J. (2023). Intraoperative Neurophysiological Monitoring.

[25] Mandeville R., Sanchez B., Johnston B., Bazarek S., Thum J.A., Birmingham A., See R.H.B., Leochico C.F.D., Kumar V., Dowlatshahi A.S. (2023). A scoping review of current and emerging techniques for evaluation of peripheral nerve health, degeneration, and regeneration: Part 1, neurophysiology. J. Neural Eng..

[26] Taskiran E., Seidel K. (2022). Current use of intraoperative neurophysiology in neurosurgery: Supratentorial part 1. Turk. Neurosurg..

[27] Pajewski T.N., Arlet V., Phillips L.H. (2007). Current approach on spinal cord monitoring: The point of view of the neurologist, the anesthesiologist and the spine surgeon. Eur. Spine J..

[28] Niu J., Ding L., Li J.J., Kim H., Liu J., Li H., Moberly A., Badea T.C., Duncan I.D., Son Y.J. (2013). Modality-based organization of ascending somatosensory axons in the direct dorsal column pathway. J. Neurosci..

[29] MacDonald D.B., Dong C., Quatrale R., Sala F., Skinner S., Soto F., Szelenyi A. (2019). Recommendations of the international society of intraoperative neurophysiology for intraoperative somatosensory evoked potentials. Clin. Neurophysiol..

[30] Wong A.K., Shils J.L., Sani S.B., Byrne R.W. (2022). Intraoperative neuromonitoring. Neurol. Clin..

[31] Szelenyi A., Langer D., Beck J., Raabe A., Flamm E.S., Seifert V., Deletis V. (2007). Transcranial and direct cortical stimulation for motor evoked potential monitoring in intracerebral aneurysm surgery. Neurophysiol. Clin..

[32] Rothwell J., Burke D., Hicks R., Stephen J., Woodforth I., Crawford M. (1994). Transcranial electrical stimulation of the motor cortex in man: Further evidence for the site of activation. J. Physiol..

[33] Olmsted Z.T., Ryu B., Phayal G., Green R., Lo S.L., Sciubba D.M., Silverstein J.W., D’Amico R.S. (2023). Direct wave intraoperative neuromonitoring for spinal tumor resection: A focused review. World Neurosurg. X.

[34] Shigematsu H., Ando M., Kobayashi K., Yoshida G., Funaba M., Morito S., Takahashi M., Ushirozako H., Kawabata S., Yamada K. (2023). Efficacy of d-wave monitoring combined with the transcranial motor-evoked potentials in high-risk spinal surgery: A retrospective multicenter study of the monitoring committee of the japanese society for spine surgery and related research. Global Spine J..

[35] Ghadirpour R., Nasi D., Iaccarino C., Romano A., Motti L., Sabadini R., Valzania F., Servadei F. (2018). Intraoperative neurophysiological monitoring for intradural extramedullary spinal tumors: Predictive value and relevance of d-wave amplitude on surgical outcome during a 10-year experience. J. Neurosurg. Spine.

[36] Kobayashi S., Matsuyama Y., Shinomiya K., Kawabata S., Ando M., Kanchiku T., Saito T., Takahashi M., Ito Z., Muramoto A. (2014). A new alarm point of transcranial electrical stimulation motor evoked potentials for intraoperative spinal cord monitoring: A prospective multicenter study from the spinal cord monitoring working group of the japanese society for spine surgery and related research. J. Neurosurg. Spine.

[37] Costa P., Peretta P., Faccani G. (2013). Relevance of intraoperative d wave in spine and spinal cord surgeries. Eur. Spine J..

[38] Kabir S.S., Jahangiri F.R., Rinesmith C., Vilches C.S., Chakarvarty S. (2023). Intraoperative testing during the mapping of the language cortex. Cureus.

[39] Gonen T., Gazit T., Korn A., Kirschner A., Perry D., Hendler T., Ram Z. (2017). Intra-operative multi-site stimulation: Expanding methodology for cortical brain mapping of language functions. PLoS ONE.

[40] Legatt A.D. (2002). Mechanisms of intraoperative brainstem auditory evoked potential changes. J. Clin. Neurophysiol..

[41] Fischer G., Fischer C., Remond J. (1992). Hearing preservation in acoustic neurinoma surgery. J. Neurosurg..

[42] Watanabe E., Schramm J., Strauss C., Fahlbusch R. (1989). Neurophysiologic monitoring in posterior fossa surgery. II. Baep-waves I and V and preservation of hearing. Acta Neurochir..

[43] Gardner G., Robertson J.H. (1988). Hearing preservation in unilateral acoustic neuroma surgery. Ann. Otol. Rhinol. Laryngol..

[44] Park S.K., Joo B.E., Lee S., Lee J.A., Hwang J.H., Kong D.S., Seo D.W., Park K., Lee H.T. (2018). The critical warning sign of real-time brainstem auditory evoked potentials during microvascular decompression for hemifacial spasm. Clin. Neurophysiol..

[45] Polo G., Fischer C., Sindou M.P., Marneffe V. (2004). Brainstem auditory evoked potential monitoring during microvascular decompression for hemifacial spasm: Intraoperative brainstem auditory evoked potential changes and warning values to prevent hearing loss—prospective study in a consecutive series of 84 patients. Neurosurgery.

[46] Legatt A.D. (2018). Electrophysiology of cranial nerve testing: Auditory nerve. J. Clin. Neurophysiol..

[47] De Moraes C.G. (2013). Anatomy of the visual pathways. J. Glaucoma.

[48] Copenhaver R.M., Beinhocker G.D. (1963). Evoked occipital potentials recorded from scalp electrodes in response to focal visual illumination. Investig. Ophthalmol..

[49] Rajashekar D., Lavrador J.P., Ghimire P., Keeble H., Harris L., Pereira N., Patel S., Beyh A., Gullan R., Ashkan K. (2022). Simultaneous motor and visual intraoperative neuromonitoring in asleep parietal lobe surgery: Dual strip technique. J. Pers. Med..

[50] Ota T., Kawai K., Kamada K., Kin T., Saito N. (2010). Intraoperative monitoring of cortically recorded visual response for posterior visual pathway. J. Neurosurg..

[51] Farrell D.F., Leeman S., Ojemann G.A. (2007). Study of the human visual cortex: Direct cortical evoked potentials and stimulation. J. Clin. Neurophysiol..

[52] Gutzwiller E.M., Cabrilo I., Radovanovic I., Schaller K., Boex C. (2018). Intraoperative monitoring with visual evoked potentials for brain surgeries. J. Neurosurg..

[53] Olmsted Z.T., Silverstein J.W., Einstein E.H., Sowulewski J., Nelson P., Boockvar J.A., D’Amico R.S. (2023). Evolution of flash visual evoked potentials to monitor visual pathway integrity during tumor resection: Illustrative cases and literature review. Neurosurg. Rev..

[54] Luo Y., Regli L., Bozinov O., Sarnthein J. (2015). Clinical utility and limitations of intraoperative monitoring of visual evoked potentials. PLoS ONE.

[55] Sasaki T., Itakura T., Suzuki K., Kasuya H., Munakata R., Muramatsu H., Ichikawa T., Sato T., Endo Y., Sakuma J. (2010). Intraoperative monitoring of visual evoked potential: Introduction of a clinically useful method. J. Neurosurg..

[56] Mattogno P.P., D’Alessandris Q.G., Rigante M., Granata G., Di Domenico M., Perotti V., Montano N., Giordano M., Chiloiro S., Doglietto F. (2023). Reliability of intraoperative visual evoked potentials (iveps) in monitoring visual function during endoscopic transsphenoidal surgery. Acta Neurochir..

[57] Silverstein J.W., Shah H.A., Greisman J.D., Dadario N.B., Barbarevech K., Park J., D’Amico R.S. (2023). Adjustable, dynamic subcortical stimulation technique for brain tumor resection: A case-series. Oper. Neurosurg..

[58] Shiban E., Krieg S.M., Obermueller T., Wostrack M., Meyer B., Ringel F. (2015). Continuous subcortical motor evoked potential stimulation using the tip of an ultrasonic aspirator for the resection of motor eloquent lesions. J. Neurosurg..

[59] Seidel K., Beck J., Stieglitz L., Schucht P., Raabe A. (2013). The warning-sign hierarchy between quantitative subcortical motor mapping and continuous motor evoked potential monitoring during resection of supratentorial brain tumors. J. Neurosurg..

[60] Silverstein J.W., Shah H.A., Unadkat P., Vilaysom S., Boockvar J.A., Langer D.J., Ellis J.A., D’Amico R.S. (2023). Short and long-term prognostic value of intraoperative motor evoked potentials in brain tumor patients: A case series of 121 brain tumor patients. J. Neurooncol..

[61] Raabe A., Beck J., Schucht P., Seidel K. (2014). Continuous dynamic mapping of the corticospinal tract during surgery of motor eloquent brain tumors: Evaluation of a new method. J. Neurosurg..

[62] Palmisciano P., Haider A.S., Balasubramanian K., Dadario N.B., Robertson F.C., Silverstein J.W., D’Amico R.S. (2022). Supplementary motor area syndrome after brain tumor surgery: A systematic review. World Neurosurg..

[63] Tarapore P.E., Tate M.C., Findlay A.M., Honma S.M., Mizuiri D., Berger M.S., Nagarajan S.S. (2012). Preoperative multimodal motor mapping: A comparison of magnetoencephalography imaging, navigated transcranial magnetic stimulation, and direct cortical stimulation. J. Neurosurg..

[64] Krieg S.M., Shiban E., Buchmann N., Gempt J., Foerschler A., Meyer B., Ringel F. (2012). Utility of presurgical navigated transcranial magnetic brain stimulation for the resection of tumors in eloquent motor areas. J. Neurosurg..

[65] Julkunen P., Saisanen L., Danner N., Niskanen E., Hukkanen T., Mervaala E., Kononen M. (2009). Comparison of navigated and non-navigated transcranial magnetic stimulation for motor cortex mapping, motor threshold and motor evoked potentials. Neuroimage.

[66] Ma C.Y., Shi J.X., Wang H.D., Hang C.H., Cheng H.L., Wu W. (2009). Intraoperative indocyanine green angiography in intracranial aneurysm surgery: Microsurgical clipping and revascularization. Clin. Neurol. Neurosurg..

[67] Dashti R., Laakso A., Niemela M., Porras M., Hernesniemi J. (2009). Microscope-integrated near-infrared indocyanine green videoangiography during surgery of intracranial aneurysms: The helsinki experience. Surg. Neurol..

[68] de Oliveira J.G., Beck J., Seifert V., Teixeira M.J., Raabe A. (2008). Assessment of flow in perforating arteries during intracranial aneurysm surgery using intraoperative near-infrared indocyanine green videoangiography. Neurosurgery.

[69] Kirk H.J., Rao P.J., Seow K., Fuller J., Chandran N., Khurana V.G. (2009). Intra-operative transit time flowmetry reduces the risk of ischemic neurological deficits in neurosurgery. Br. J. Neurosurg..

[70] Kapsalaki E.Z., Lee G.P., Robinson J.S., Grigorian A.A., Fountas K.N. (2008). The role of intraoperative micro-doppler ultrasound in verifying proper clip placement in intracranial aneurysm surgery. J. Clin. Neurosci..

[71] Amin-Hanjani S., Meglio G., Gatto R., Bauer A., Charbel F.T. (2006). The utility of intraoperative blood flow measurement during aneurysm surgery using an ultrasonic perivascular flow probe. Neurosurgery.

[72] Stendel R., Pietila T., Al Hassan A.A., Schilling A., Brock M. (2000). Intraoperative microvascular doppler ultrasonography in cerebral aneurysm surgery. J. Neurol. Neurosurg. Psychiatry.

[73] Klopfenstein J.D., Spetzler R.F., Kim L.J., Feiz-Erfan I., Han P.P., Zabramski J.M., Porter R.W., Albuquerque F.C., McDougall C.G., Fiorella D.J. (2004). Comparison of routine and selective use of intraoperative angiography during aneurysm surgery: A prospective assessment. J. Neurosurg..

[74] Chappell E.T., Moure F.C., Good M.C. (2003). Comparison of computed tomographic angiography with digital subtraction angiography in the diagnosis of cerebral aneurysms: A meta-analysis. Neurosurgery.

[75] Chiang V.L., Gailloud P., Murphy K.J., Rigamonti D., Tamargo R.J. (2002). Routine intraoperative angiography during aneurysm surgery. J. Neurosurg..

[76] Miller D.R., Ashour R., Sullender C.T., Dunn A.K. (2022). Continuous blood flow visualization with laser speckle contrast imaging during neurovascular surgery. Neurophotonics.

[77] Nomura S., Inoue T., Ishihara H., Koizumi H., Suehiro E., Oka F., Suzuki M. (2014). Reliability of laser speckle flow imaging for intraoperative monitoring of cerebral blood flow during cerebrovascular surgery: Comparison with cerebral blood flow measurement by single photon emission computed tomography. World Neurosurg..

[78] Hecht N., Woitzik J., Konig S., Horn P., Vajkoczy P. (2013). Laser speckle imaging allows real-time intraoperative blood flow assessment during neurosurgical procedures. J. Cereb. Blood Flow. Metab..

[79] Hecht N., Woitzik J., Dreier J.P., Vajkoczy P. (2009). Intraoperative monitoring of cerebral blood flow by laser speckle contrast analysis. Neurosurg. Focus.

[80] Calderon-Arnulphi M., Alaraj A., Amin-Hanjani S., Mantulin W.W., Polzonetti C.M., Gratton E., Charbel F.T. (2007). Detection of cerebral ischemia in neurovascular surgery using quantitative frequency-domain near-infrared spectroscopy. J. Neurosurg..

[81] Debatisse D., Pralong E., Dehdashti A.R., Regli L. (2005). Simultaneous multilobar electrocorticography (mecog) and scalp electroencephalography (scalp eeg) during intracranial vascular surgery: A new approach in neuromonitoring. Clin. Neurophysiol..

[82] Dehdashti A.R., Pralong E., Debatisse D., Regli L. (2006). Multilobar electrocorticography monitoring during intracranial aneurysm surgery. Neurocrit. Care.

[83] Bacigaluppi S., Fontanella M., Manninen P., Ducati A., Tredici G., Gentili F. (2012). Monitoring techniques for prevention of procedure-related ischemic damage in aneurysm surgery. World Neurosurg..

[84] Young W.L., Solomon R.A., Pedley T.A., Ross L., Schwartz A.E., Ornstein E., Matteo R.S., Ostapkovich N. (1989). Direct cortical eeg monitoring during temporary vascular occlusion for cerebral aneurysm surgery. Anesthesiology.

[85] Carter L.P., Raudzens P.A., Gaines C., Crowell R.M. (1984). Somatosensory evoked potentials and cortical blood flow during craniotomy for vascular disease. Neurosurgery.

[86] Lee S.K. (2023). Who are the better candidates for epilepsy surgery?. J. Epilepsy Res..

[87] Stone S.S., Rutka J.T. (2008). Utility of neuronavigation and neuromonitoring in epilepsy surgery. Neurosurg. Focus.

[88] Sugano H., Shimizu H., Sunaga S. (2007). Efficacy of intraoperative electrocorticography for assessing seizure outcomes in intractable epilepsy patients with temporal-lobe-mass lesions. Seizure.

[89] Szelenyi A., Joksimovic B., Seifert V. (2007). Intraoperative risk of seizures associated with transient direct cortical stimulation in patients with symptomatic epilepsy. J. Clin. Neurophysiol..

[90] Moiyadi A., Velayutham P., Shetty P., Seidel K., Janu A., Madhugiri V., Singh V.K., Patil A., John R. (2018). Combined motor evoked potential monitoring and subcortical dynamic mapping in motor eloquent tumors allows safer and extended resections. World Neurosurg..

[91] Berg A.T., Vickrey B.G., Langfitt J.T., Sperling M.R., Walczak T.S., Shinnar S., Bazil C.W., Pacia S.V., Spencer S.S. (2003). The multicenter study of epilepsy surgery: Recruitment and selection for surgery. Epilepsia.

[92] Yang T., Hakimian S., Schwartz T.H. (2014). Intraoperative electrocorticography (ecog): Indications, techniques, and utility in epilepsy surgery. Epileptic Disord..

[93] Freund B.E., Feyissa A.M., Khan A., Middlebrooks E.H., Grewal S.S., Sabsevitz D., Sherman W.J., Quinones-Hinojosa A., Tatum W.O. (2024). Early postoperative seizures following awake craniotomy and functional brain mapping for lesionectomy. World Neurosurg..

[94] Tang L., Tan T.K. (2024). Anaesthetic considerations and challenges during awake craniotomy. Singapore Med. J..

[95] Isnard J., Taussig D., Bartolomei F., Bourdillon P., Catenoix H., Chassoux F., Chipaux M., Clemenceau S., Colnat-Coulbois S., Denuelle M. (2018). French guidelines on stereoelectroencephalography (seeg). Neurophysiol. Clin..

[96] Reames D.L., Smith J.S., Fu K.M., Polly D.W., Ames C.P., Berven S.H., Perra J.H., Glassman S.D., McCarthy R.E., Knapp R.D. (2011). Complications in the surgical treatment of 19,360 cases of pediatric scoliosis: A review of the scoliosis research society morbidity and mortality database. Spine.

[97] Lavano A., Della Torre A., Guzzi G., Domenico L.T. (2023). Plica mediana dorsalis as a potential risk for spine surgery. J. Neurosurg. Sci..

[98] Vitale M.G., Moore D.W., Matsumoto H., Emerson R.G., Booker W.A., Gomez J.A., Gallo E.J., Hyman J.E., Roye D.P. (2010). Risk factors for spinal cord injury during surgery for spinal deformity. J. Bone Jt. Surg. Am..

[99] Fehlings M.G., Brodke D.S., Norvell D.C., Dettori J.R. (2010). The evidence for intraoperative neurophysiological monitoring in spine surgery: Does it make a difference?. Spine.

[100] Breinin G.M., Sadovnikoff N., Pfeffer R., Davidowitz J., Chiarandini D.J. (1985). Cadmium reduces extraocular muscle contractility in vitro and in vivo. Investig. Ophthalmol. Vis. Sci..

[101] Langeloo D.D., Lelivelt A., Louis Journee H., Slappendel R., de Kleuver M. (2003). Transcranial electrical motor-evoked potential monitoring during surgery for spinal deformity: A study of 145 patients. Spine.

[102] Pelosi L., Lamb J., Grevitt M., Mehdian S.M., Webb J.K., Blumhardt L.D. (2002). Combined monitoring of motor and somatosensory evoked potentials in orthopaedic spinal surgery. Clin. Neurophysiol..

[103] Chandra A.A., Vaishnav A., Shahi P., Song J., Mok J., Alluri R.K., Chen D., Gang C.H., Qureshi S. (2023). The role of intraoperative neuromonitoring modalities in anterior cervical spine surgery. HSS J..

[104] Costa F., Anania C.D., Agrillo U., Roberto A., Claudio B., Simona B., Daniele B., Carlo B., Barbara C., Ardico C. (2023). Cervical spondylotic myelopathy: From the world federation of neurosurgical societies (wfns) to the italian neurosurgical society (sinch) recommendations. Neurospine.

[105] Bajamal A.H., Kim S.H., Arifianto M.R., Faris M., Subagio E.A., Roitberg B., Udo-Inyang I., Belding J., Zileli M., Parthiban J. (2019). Posterior surgical techniques for cervical spondylotic myelopathy: Wfns spine committee recommendations. Neurospine.

[106] Deora H., Kim S.H., Behari S., Rudrappa S., Rajshekhar V., Zileli M., Parthiban J. (2019). Anterior surgical techniques for cervical spondylotic myelopathy: Wfns spine committee recommendations. Neurospine.

[107] Zileli M., Borkar S.A., Sinha S., Reinas R., Alves O.L., Kim S.H., Pawar S., Murali B., Parthiban J. (2019). Cervical spondylotic myelopathy: Natural course and the value of diagnostic techniques -wfns spine committee recommendations. Neurospine.

[108] Zileli M. (2019). Recommendations of wfns spine committee. Neurospine.

[109] Funaba M., Kanchiku T., Yoshida G., Machino M., Ushirozako H., Kawabata S., Ando M., Yamada K., Iwasaki H., Shigematsu H. (2023). Impact of preoperative motor status for the positive predictive value of transcranial motor-evoked potentials alerts in thoracic spine surgery: A prospective multicenter study by the monitoring committee of the japanese society for spine surgery and related research. Global Spine J..

[110] D’Ercole M., D’Alessandris Q.G., Di Domenico M., Burattini B., Menna G., Izzo A., Polli F.M., Della Pepa G.M., Olivi A., Montano N. (2023). Is there a role for intraoperative neuromonitoring in intradural extramedullary spine tumors? Results and indications from an institutional series. J. Pers. Med..

[111] Hockel J.L. (1980). The face bow: A primary diagnostic aid to gaining an organic occlusion, the goal of orthopedic gnathology. Int. J. Orthod..

[112] Rijs K., Klimek M., Scheltens-de Boer M., Biesheuvel K., Harhangi B.S. (2019). Intraoperative neuromonitoring in patients with intramedullary spinal cord tumor: A systematic review, meta-analysis, and case series. World Neurosurg..

[113] van der Wal E.C., Klimek M., Rijs K., Scheltens-de Boer M., Biesheuvel K., Harhangi B.S. (2021). Intraoperative neuromonitoring in patients with intradural extramedullary spinal cord tumor: A single-center case series. World Neurosurg..

[114] Ushirozako H., Yoshida G., Imagama S., Machino M., Ando M., Kawabata S., Yamada K., Kanchiku T., Fujiwara Y., Taniguchi S. (2023). Role of transcranial motor evoked potential monitoring during traumatic spinal injury surgery: A prospective multicenter study of the monitoring committee of the Japanese society for spine surgery and related research. Spine.

[115] Ajiboye R.M., Zoller S.D., Sharma A., Mosich G.M., Drysch A., Li J., Reza T., Pourtaheri S. (2017). Intraoperative neuromonitoring for anterior cervical spine surgery: What is the evidence?. Spine.

[116] Atesok K., Smith W., Jones R., Niemeier T., Manoharan S.R.R., McGwin G., Pittman J., Theiss S. (2018). The significance of upper extremity neuromonitoring changes during thoracolumbar spine surgery. Clin. Spine Surg..

[117] Pusat S., Kural C., Solmaz I., Temiz C., Kacar Y., Tehli O., Kutlay M., Daneyemez M., Izci Y. (2017). Comparison of electrophysiological outcomes of tethered cord syndrome and spinal intradural tumors: A retrospective clinical study. Turk. Neurosurg..

[118] Park J.H., Lee S.H., Kim E.S., Eoh W. (2018). Analysis of multimodal intraoperative monitoring during intramedullary spinal ependymoma surgery. World Neurosurg..

[119] Gruenbaum B.F., Gruenbaum S.E. (2019). Neurophysiological monitoring during neurosurgery: Anesthetic considerations based on outcome evidence. Curr. Opin. Anaesthesiol..

[120] James G. (1987). Mental handicap: As the tide turns. Community Outlook.

[121] Guo L., Holdefer R.N., Kothbauer K.F. (2022). Monitoring spinal surgery for extramedullary tumors and fractures. Handb. Clin. Neurol..

[122] Skinner S., Guo L. (2022). Intraoperative neuromonitoring during surgery for lumbar stenosis. Handb. Clin. Neurol..

[123] Visalli C., Cavallaro M., Concerto A., La Torre D., Di Salvo R., Mazziotti S., Salamone I. (2018). Ultrasonography of traumatic injuries to limb peripheral nerves: Technical aspects and spectrum of features. Jpn. J. Radiol..

[124] La Torre D., Raffa G., Pino M.A., Fodale V., Rizzo V., Visalli C., Guzzi G., Della Torre A., Lavano A., Germano A. (2018). A novel diagnostic and prognostic tool for simple decompression of ulnar nerve in cubital tunnel syndrome. World Neurosurg..

[125] Xiao B., Constatntini S., Browd S.R., Zhan Q., Jiang W., Mei R. (2020). The role of intra-operative neuroelectrophysiological monitoring in single-level approach selective dorsal rhizotomy. Childs Nerv. Syst..

[126] Noland S.S., Bishop A.T., Spinner R.J., Shin A.Y. (2019). Adult traumatic brachial plexus injuries. J. Am. Acad. Orthop. Surg..

[127] Anderson J.C., Yamasaki D.S. (2016). Intraoperative nerve monitoring during nerve decompression surgery in the lower extremity. Clin. Podiatr. Med. Surg..

[128] Plata-Bello J., Perez-Lorensu P.J., Brage L., Hernandez-Hernandez V., Doniz A., Roldan-Delgado H., Febles P., Garcia-Conde M., Perez-Orribo L., Garcia-Marin V. (2015). Electrical stimulation threshold in chronically compressed lumbar nerve roots: Observational study. Clin. Neurol. Neurosurg..

[129] Kurup A.N., Morris J.M., Boon A.J., Strommen J.A., Schmit G.D., Atwell T.D., Carter R.E., Brown M.J., Wass C.T., Rose P.S. (2014). Motor evoked potential monitoring during cryoablation of musculoskeletal tumors. J. Vasc. Interv. Radiol..

[130] Fernandez-Conejero I., Ulkatan S., Sen C., Deletis V. (2012). Intra-operative neurophysiology during microvascular decompression for hemifacial spasm. Clin. Neurophysiol..

[131] Sughrue M.E., Yang I., Rutkowski M.J., Aranda D., Parsa A.T. (2010). Preservation of facial nerve function after resection of vestibular schwannoma. Br. J. Neurosurg..

[132] Abrosimov V.N., Garmash V. (1988). The hyperventilation syndrome. Ter. Arkh.

[133] Zelenski N.A., Oishi T., Shin A.Y. (2023). Intraoperative neuromonitoring for peripheral nerve surgery. J. Hand Surg. Am..

[134] Robert E.G., Happel L.T., Kline D.G. (2009). Intraoperative nerve action potential recordings: Technical considerations, problems, and pitfalls. Neurosurgery.

[135] Nunes R.R., Bersot C.D.A., Garritano J.G. (2018). Intraoperative neurophysiological monitoring in neuroanesthesia. Curr. Opin. Anaesthesiol..

[136] Prathapadas U., Hrishi A.P., Appavoo A., Vimala S., Sethuraman M. (2020). Effect of low-dose dexmedetomidine on the anesthetic and recovery profile of sevoflurane-based anesthesia in patients presenting for supratentorial neurosurgeries: A randomized double-blind placebo-controlled trial. J. Neurosci. Rural. Pract..

[137] Teixeira M.T., Brinkman N.J., Pasternak J.J., Abcejo A.S. (2024). The role of remimazolam in neurosurgery and in patients with neurological diseases: A narrative review. J. Neurosurg. Anesthesiol..

[138] Eisenried A., Schuttler J., Lerch M., Ihmsen H., Jeleazcov C. (2020). Pharmacokinetics and pharmacodynamics of remimazolam (cns 7056) after continuous infusion in healthy male volunteers: Part ii. Pharmacodynamics of electroencephalogram effects. Anesthesiology.

[139] Shirozu K., Nobukuni K., Tsumura S., Imura K., Nakashima K., Takamori S., Higashi M., Yamaura K. (2022). Neurological sedative indicators during general anesthesia with remimazolam. J. Anesth..

[140] Tanaka R., Sato A., Shinohara K., Shiratori T., Kiuchi C., Murakami T., Sasao J. (2022). Comparison of sensory evoked potentials during neurosurgery under remimazolam anesthesia with those under propofol anesthesia. Minerva Anestesiol..

[141] Kondo T., Toyota Y., Narasaki S., Watanabe T., Miyoshi H., Saeki N., Tsutsumi Y.M. (2020). Intraoperative responses of motor evoked potentials to the novel intravenous anesthetic remimazolam during spine surgery: A report of two cases. JA Clin. Rep..

[142] Zhang X., Hu H., Yan R., Li T., Wang W., Yang W. (2022). Effects of rocuronium dosage on intraoperative neurophysiological monitoring in patients undergoing spinal surgery. J. Clin. Pharm. Ther..

[143] Namizato D., Iwasaki M., Ishikawa M., Nagaoka R., Genda Y., Kishikawa H., Sugitani I., Sakamoto A. (2019). Anesthetic considerations of intraoperative neuromonitoring in thyroidectomy. J. Nippon. Med. Sch..

[144] Han Y.D., Liang F., Chen P. (2015). Dosage effect of rocuronium on intraoperative neuromonitoring in patients undergoing thyroid surgery. Cell Biochem. Biophys..

[145] Lu I.C., Tsai C.J., Wu C.W., Cheng K.I., Wang F.Y., Tseng K.Y., Chiang F.Y. (2011). A comparative study between 1 and 2 effective doses of rocuronium for intraoperative neuromonitoring during thyroid surgery. Surgery.

[146] Empis de Vendin O., Schmartz D., Brunaud L., Fuchs-Buder T. (2017). Recurrent laryngeal nerve monitoring and rocuronium: A selective sugammadex reversal protocol. World J. Surg..

[147] Fahy B.G., Chau D.F. (2018). The technology of processed electroencephalogram monitoring devices for assessment of depth of anesthesia. Anesth. Analg..

[148] Longhini F., Pasin L., Montagnini C., Konrad P., Bruni A., Garofalo E., Murabito P., Pelaia C., Rondi V., Dellapiazza F. (2021). Intraoperative protective ventilation in patients undergoing major neurosurgical interventions: A randomized clinical trial. BMC Anesthesiol..

[149] Citerio G., Pesenti A., Latini R., Masson S., Barlera S., Gaspari F., Franzosi M.G. (2012). A multicentre, randomised, open-label, controlled trial evaluating equivalence of inhalational and intravenous anaesthesia during elective craniotomy. Eur. J. Anaesthesiol..

[150] Lotto M.L., Banoub M., Schubert A. (2004). Effects of anesthetic agents and physiologic changes on intraoperative motor evoked potentials. J. Neurosurg. Anesthesiol..

[151] Sloan T.B., Heyer E.J. (2002). Anesthesia for intraoperative neurophysiologic monitoring of the spinal cord. J. Clin. Neurophysiol..

[152] Adhikary S.D., Manickam B.P. (2008). Unusual waveforms during ssep monitoring--facts and artifacts. J. Neurosurg. Anesthesiol..

[153] Scott R.N., McLean L., Parker P.A. (1997). Stimulus artefact in somatosensory evoked potential measurement. Med. Biol. Eng. Comput..

[154] Sloan T.B. (1994). Nondepolarizing neuromuscular blockade does not alter sensory evoked potentials. J. Clin. Monit..

[155] Farajidavar A., Seifert J.L., Delgado M.R., Sparagana S., Romero-Ortega M.I., Chiao J.C. (2016). Electromagnetic interference in intraoperative monitoring of motor evoked potentials and a wireless solution. Med. Eng. Phys..

[156] Gonzalez A.A., Akopian V., Lagoa I., Shilian P., Parikh P. (2019). Crossover phenomena in motor evoked potentials during intraoperative neurophysiological monitoring of cranial surgeries. J. Clin. Neurophysiol..

[157] Delgado-Lopez P.D., Montalvo-Afonso A., Araus-Galdos E., Isidro-Mesa F., Martin-Alonso J., Martin-Velasco V., Castilla-Diez J.M., Rodriguez-Salazar A. (2022). Need for head and neck repositioning to restore electrophysiological signal changes at positioning for cervical myelopathy surgery. Neurocirugia.

[158] Appel S., Korn A., Biron T., Goldstein K., Rand N., Millgram M., Floman Y., Ashkenazi E. (2017). Efficacy of head repositioning in restoration of electrophysiological signals during cervical spine procedures. J. Clin. Neurophysiol..

