# Supplementary material for: Intraoperative Neurophysiological Monitoring in Neurosurgery
Source: J Clin Med. 2024 May 17;13(10):2966. doi: 10.3390/jcm13102966 (PMC11122101; doi:10.3390/jcm13102966)
Supplement: Supplementary file 1 [file jcm-13-02966-s001.zip › jcm-2991950-supplementary.pdf]

INTRAOPERATIVE NEUROPHYSIOLOGICAL MONITORING IN NEUROSURGERY

Giusy Guzzi, Riccardo Antonio Ricciuti, Attilio Della Torre, Erica Lo Turco, Angelo Lavano, Federico Longhini and Domenico La Torre.

SUPPLEMENTARY MATERIALS

SEARCH STRATEGY

(“Intraoperative Neurophysiological Monitoring” [MeSH] OR “electrocorticography” [MeSH] OR “electromyography” [MeSH] OR “Evoked Potentials” [MeSH] OR “Intraoperative Neurophysiological Monitoring” [All fields] OR “neuromonitoring” [All fields] OR “electrocorticography” [All fields] OR “Electrocorticographies” [All fields] OR “ECoG” [All fields] OR “stereo-electroencephalography” [All fields] OR “electromyography” [All fields] OR “EMG” [All fields] OR “Electromyogram” [All fields] OR “somatosensory evoked potentials” [All fields] OR “SSEP” [All fields] OR “motor evoked potential” [All fields] OR “MEP” [All fields] OR “brainstem auditory evoked potentials” [All fields] OR “BAEP” [All fields] OR “auditory evoked potentials” [All fields] OR “visual evoked potentials” [All fields] OR “VEP” [All fields] OR “direct cortical stimulation” [All fields]) AND (“Neurosurgery” [MeSH] OR “Neurosurgical procedure” [MeSH] OR “Neurosurgery” [All fields] OR “Neurosurgical Procedure” [All fields] OR “Spinal Surgery” [All fields] OR “Peripheral Nerve Surgery” [All fields] OR “Brain surgery” [All fields])

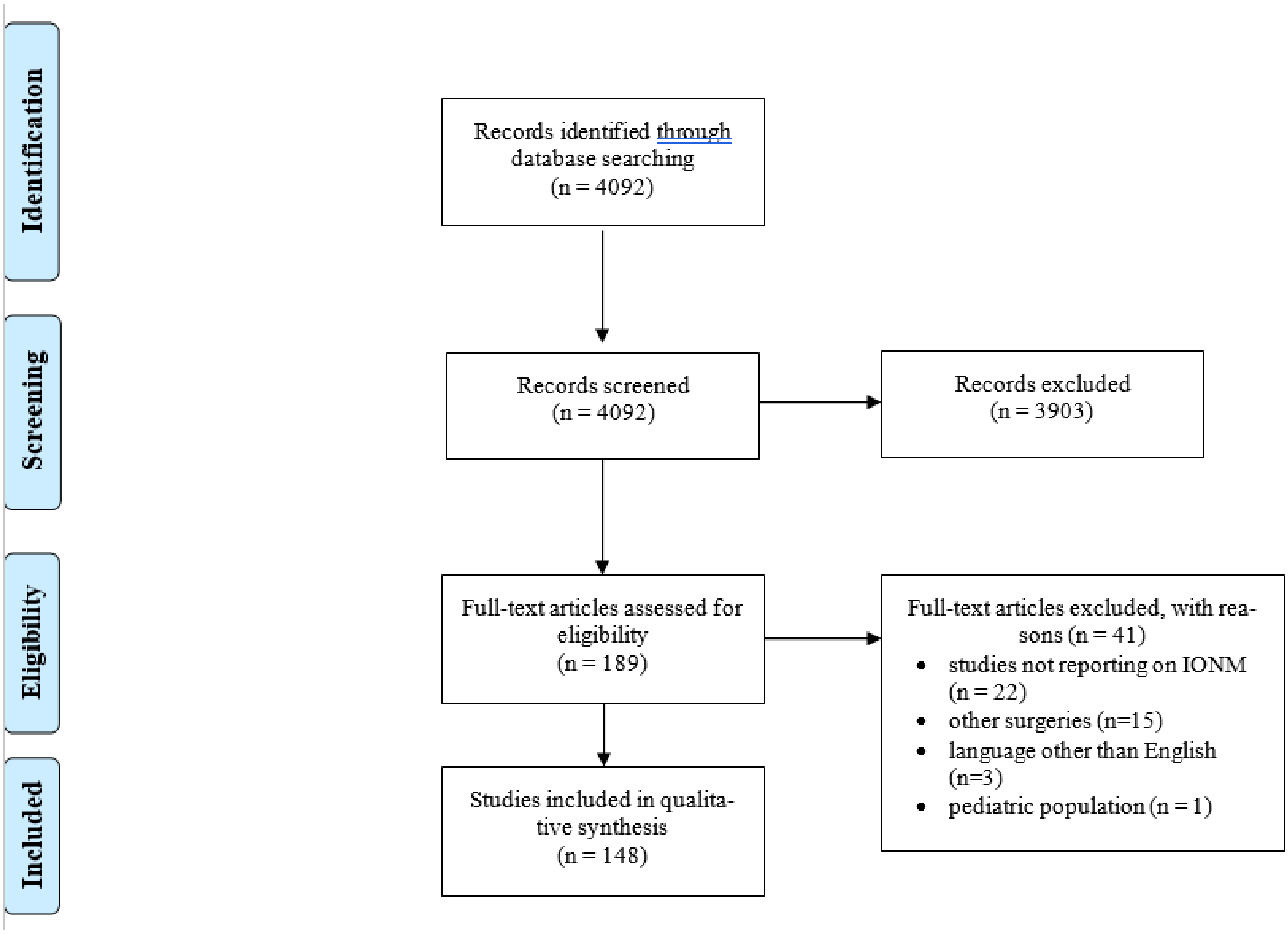

FIGURE S1. FLOW DIAGRAM.

Study flow diagram according to the Preferred Reporting Items for Systematic review and Meta-Analysis Protocols recommendations.
